# Dimensions of alexithymia and their links to anxiety and depression

**DOI:** 10.1192/j.eurpsy.2022.1168

**Published:** 2022-09-01

**Authors:** M. Ben Abdallah, I. Baati, F. Tabib, F. Guermazi, S. Hentati, N. Farhat, J. Masmoudi

**Affiliations:** 1 CHU Hedi CHaker hospital Sfax Tunisia, Department Of Psychiatry (a), Sfax, Tunisia; 2 CHU Hedi CHaker hospital Sfax Tunisia, Department Of Psychiatry (a), sfax, Tunisia; 3 Habib Bourguiba University Hospital, Department Of Neurology, Sfax, Tunisia

**Keywords:** Dimensions of alexithymia, Depression, Anxiety

## Abstract

**Introduction:**

Anxiety and depression are among the most common psychiatric comorbidities in multiple sclerosis (MS) patients. These disorders could lead to significant emotional disturbances.

**Objectives:**

To study the different dimensions of alexithymia in patients with MS and determine their relationship with anxiety and depression.

**Methods:**

Our study, descriptive and analytical, focused on patients followed for MS at the neurology department in Sfax (Tunisia). In addition to collecting sociodemographic data, we used the Hospital Anxiety and Depression Scale (HADS) to assess anxiety and depressive symptoms and the Toronto Alexithymia Scale (TAS-20) to assess alexithymia and its three dimensions: difficulty identifying emotions (DIE), difficulty differentiating emotions (DDE), and externally oriented thinking (EOT).

**Results:**

This study included 93 patients followed for MS. Our results showed a prevalence of 58.1% for alexithymia, 38.7% for anxiety and 26.9% for depression. The median score of the dimension DIE was 22. The median score of the dimension DDE was 17. The mean score for the dimension EOT was 26.96 ± 4.18. Alexithymic patients were more anxious and depressed (p = 0,002 and p < 10^-3^, respectively). Both dimensions DIE and DDE were associated with anxiety (p = 0.001 and p = 0.022, respectively) and depression (p < 10^-3^ and p < 10^-3^, respectively). Non-depressed patients had a higher score on the EOT dimension (p = 0.003).

**Conclusions:**

Our results showed a relationship between depression, anxiety and alexithymia, hence the importance of looking for alexithymia in MS patients with anxiety or depressive symptoms.

**Disclosure:**

No significant relationships.

